# Cone beam CT pre‐ and post‐daily treatment for assessing geometrical and dosimetric intrafraction variability during radiotherapy of prostate cancer

**DOI:** 10.1120/jacmp.v12i1.3371

**Published:** 2010-12-02

**Authors:** Giacomo Reggiori, Pietro Mancosu, Angelo Tozzi, Marie C Cantone, Simona Castiglioni, Paola Lattuada, Francesca Lobefalo, Luca Cozzi, Antonella Fogliata, Piera Navarria, Marta Scorsetti

**Affiliations:** ^1^ Radiation Oncology Dept. IRCCS Istituto Clinico Humanitas Milano (Rozzano) Italy; ^2^ Physics Dept. Università degli studi di Milano Milano Italy; ^3^ Medical Physics Unit Oncology Institute of Southern Switzerland Bellinzona Switzerland

**Keywords:** CBCT, RapidArc, prostate RT, intrafraction motion

## Abstract

The purpose of this study was to quantify the relationship between treatment time and dose uncertainty due to intrafraction organ motion in prostate cancer radiotherapy (RT). Ten consecutive patients with prostate cancer treated by radical RT by volumetric modulated arc therapy (RapidArc) were considered. For each patient, pre‐ and post‐treatment cone beam computed tomography (CBCT) was performed in 10 fractions. The prostate, rectum and bladder were contoured on each CBCT. The change in organ position, volume and dosimetric uncertainty induced by organ motion were evaluated. Interval time between the two CBCTs ranged between 4 and 16 min (mean 7.3±0.7 min). Treatment with intrafraction prostate motion > 3 mm and > 5 mm were 24% and 5%, respectively. Regarding change in centroid position and volume, a poor time correlation was found for target and rectum, while a constant increase was obtained for bladder. The agreement index was highly correlated to time (r=−0.89 for bladder, r=−0.95 for rectum, and r=−0.84 for prostate). In terms of difference in dose volume histogram between pre‐ and post‐CBCT, the dose uncertainties for the targets and rectum amplified with the increasing time. The increasing intrafraction dose uncertainty with time requires the use of an RT technique with minimization of treatment time to improve confidence in planning dose distribution.

PACS number: 87.55.tm

## I. INTRODUCTION

Prostate tumor is the most commonly diagnosed male cancer worldwide. Radiotherapy (RT) has been shown to allow for good local control and very few side‐effects. Literature data show improved tumor control with the use of higher‐doses of RT. This has been possible with the advent of three‐dimensional conformal RT (3D‐CRT) that allows dose escalation to the prostate and concomitant sparing of the surrounding rectum and bladder. With the development of even more sophisticated treatment‐planning software and multileaf collimators, intensity‐modulated radiation therapy (IMRT) emerged as an advanced form of shaped technique.

Anatomically, the prostate is located between the rectum and bladder and both are affected by physiological changes, in particular shape and size. These are not related to pelvic bone anatomy. These changes can induce prostate dislocation during RT courses at both intra‐ and interfraction level. As a consequence, possible underdosage of target or overdosage of organs at risk (OAR) might arise. To avoid the former, margins around the clinical target volume (CTV) are considered to include the target during each RT fraction, producing the planning target volume (PTV) over which the dose is prescribed. In order to reduce toxicity to OAR, methods to reduce CTV to PTV margins without compromising target coverage are required, particularly when high doses are prescribed.

Image‐guided RT (IGRT) with verification of the organ position before the daily treatment has allowed for lessening of interfraction target position modifications^(^ ^1^ ^,^ ^2^ ^)^ and for substantial reduction of target margins. In this context, linac vendors have recently made available a kV cone beam CT (CBCT) based on flat‐panel technology integrated with a medical linear accelerator for therapy guidance. The volumetric images may be used to verify and correct the planning patient setup in the linac coordinates by comparing these with the patient position defined in the treatment plan.

Recent studies analyzed pre‐ and post‐treatment CBCT^(^ ^3^ ^–^ ^5^ ^)^ for evaluation of intrafractional motion during intensity‐modulated RT (IMRT) with a fixed gantry.

RapidArc (Varian Medical Systems, Palo Alto, CA) is the Varian solution for delivering volumetric modulated arc therapy (VMAT). Many groups compared this new technique to intensity‐modulated RT (IMRT) with static gantry, showing better target coverage and OAR sparing for many organ regions, with lower field on time.^(^ ^6^ ^–^ ^10^ ^)^

The aim of the present investigation was to evaluate dependence on time of the intrafraction organ motion by performing CBCT immediately pre‐ and post‐treatment. The starting hypothesis was that the shorter the delivery time, the lesser the organ deformation and dislocation. In particular, a specific analysis was performed for assessing the intrafraction discrepancy in dose.

## II. MATERIALS AND METHODS

### A. Patient selection

Ten consecutive patients with low to medium risk, histologically proven, prostate adenocarcinoma were considered in this study. RT was prescribed to all patients and, as part of a routine protocol, patients were instructed to empty the rectum by enema and to void the bladder one hour before the daily treatment, and then drink 0.5 L of water without empting the bladder until treatment is over. No specific diet instructions were given.

### B. Simulation and planning

Patients were scanned in supine position by 16 slice CT with a slice thickness of 3 mm. Clinical target volume (CTV) was defined as the prostate plus seminal vesicles; CTV1 was defined as the CTV excluding the seminal vesicles (i.e., prostate alone), while CTV2 was defined as the seminal vesicles alone. The planning target volume (PTV) was defined as CTV + 8 mm, except for the cranial caudal direction where an expansion of 10 mm was considered. The isocenter was defined on virtual CT simulation as the CTV center of mass. A standard protocol was adopted, prescribing 76 Gy in fractions of 2 Gy/day (i.e., 38 fractions) to mean PTV1. The prescribed dose to mean PTV2 was 68 Gy (1.8 Gy/day). All treatments were planned according to the simultaneous integrated boost method. Bladder, rectum and femoral heads were the OAR considered. Plan objectives were: PTV1 and PTV2: $V_{95\%}>95\%$ (at least 95% of the PTV volume must be covered by 95% of the prescribed dose); $D_{1\%}<107\%$ (less than 1% of the volume should receive 107% of the prescribed dose). For OARs, the following constrains were considered: for bladder, $D_{\text{Max}}<76$ Gy (maximal dose to the bladder lower than 76 Gy); for rectum, $D_{\text{Max}}<76\;\text{Gy},\;V_{75\text{Gy}}<5\%$ (rectal volume receiving more than 75 Gy less than 5%), $V_{70\text{Gy}}<25\%,V_{60\text{Gy}}<40\%$; for femoral heads, $V_{45\text{Gy}}<10\%$.^(11)^ All plans were optimized by RapidArc technique, using a single partial arc (from 220° up to +140°) for a 6 MV photon beam.

### C. Treatment

The treatment was delivered using a Varian 2100DHX linear accelerator (Varian Medical System, Palo Alto, CA) integrated with an on‐board imager (OBI) for acquiring CBCT images. CBCT acquisition time was around 60 s and 30 s were additionally necessary by the image reconstruction engine to generate the final dataset. Reconstructed slice thickness was 3 mm, as for the simulation CT. The CBCT scans were all acquired with 120 kV, 80 mA, 13 ms for each projection; half‐fan mode was used due to the large volume investigated. Along the cranial–caudal direction, 30 cm were scanned containing the whole rectum and bladder.

On each of the first five days and then once a week during the remaining course of treatment, a CBCT of the prostate region was acquired before the treatment (${\text{CBCT}}_{I}$) after initial patient positioning. By application of a simple choice generator, the various ${\text{CBCT}}_{I}$ were randomly analyzed by a radiation oncologist who was required to perform an online co‐registration and approve the eventual couch shift according to the registration results. (This two‐path approach was initially decided upon in order to allow for quantification of the mean time necessary for the online co‐registration and evaluate its dosimetric fallout compared to the non–co‐registered fractions. The results obtained were not particularly significant and were therefore omitted in this study). This shift was applied if displacements larger than 2 mm in any direction were found. Next in the protocol order was delivery of the treatment. Immediately after completion, acquisition of a second CBCT (${\text{CBCT}}_{\text{II}}$) for off‐line analysis was carried out. In the case of no online co‐registration, the radiation oncologist in charge of reviewing the data performed an off‐line co‐registration and recorded the theoretical shift.

The time between the start of the ${\text{CBCT}}_{I}$ and the end of the ${\text{CBCT}}_{\text{II}}$ (without considering the time of reconstruction of the second ${\text{CBCT}}_{\text{II}}$) was recorded from the time line data registered by the electronic recording and verification system.

### D. Data analysis

Data for evaluation were based on 160 CBCT datasets.

In both fractions with online or with off‐line co‐registration of the ${\text{CBCT}}_{I}$ to the simulation CT, the ${\text{CBCT}}_{\text{II}}$ was automatically registered to the ${\text{CBCT}}_{I}$. This was done because by means of the second CBCT, we aimed to observe the intrafraction displacement and not the shifts from the simulation CT.

One radiation oncologist outlined the contours of CTV1, CTV2, bladder and rectum on each reconstructed slice for each CBCT using a fixed method to define the contours – in particular, contours relating to the border slices (affected by higher uncertainties). This procedure was intended to eliminate interobserver variability.

For each organ/target, volume and center of mass (COM) coordinates were calculated for all the CBCT series for geometrical analysis.

In our department, we have not yet the means to get real‐time tracking of the prostate but pre‐ and post‐treatment imaging were shown to have a good accuracy for prostate localization.^(^ ^4^ ^)^ We evaluated a linear trajectory model assessing the accuracy of using post‐treatment localization as the maximum displacement at MV delivery at 95% within 3.1 mm. Further studies including real tracking are forthcoming; however, these literature data demonstrate the acceptability of this methodology.

The COM shifts and volume variations between ${\text{CBCT}}_{I}$ and ${\text{CBCT}}_{\text{II}}$ were considered.

The COM shift was calculated for the three directions, and the distance was calculated as:

(1) $$\Delta d=\sqrt{{\left(x_{I}-x_{II}\right)}^{2}+{\left(y_{I}-y_{II}\right)}^{2}+{\left(z_{I}-z_{II}\right)}^{2}}$$

Regarding the volumes, two parameters were considered: (a) the percent difference between the organ volumes evaluated on the two CBCTs, and (b) the agreement index (AI) defined as:

(2) $$AI=\frac{I\;(V_{I},V_{II})}{U\;(V_{I},V_{II})}$$

where $V_{I}$ and $V_{\text{II}}$ are the organ volume defined on ${\text{CBCT}}_{I}$ and ${\text{CBCT}}_{\text{II}}$, respectively.

Furthermore, the intravariability in contouring delineation was evaluated by performing a second contour on 20 CBCTs randomly chosen by the dataset, and assessing the intravariability as the percentage difference in volume between the first and the second contouring. The total uncertainty for each organ considered was obtained by evaluating the mean uncertainty over the 20 CBCTs.

To complement the geometrical assessment, the treatment plans originally computed on the simulation CT data were recalculated using the daily pre‐ and post‐treatment CBCT similarly to what has been described previously by Yang et al.^(^ ^12^ ^)^ In particular, the values of $V_{90\%}$, $V_{95\%}$ for CTV1 and CTV2, and $V_{75\text{Gy}}$ (dose received more than 75 Gy) for rectum were analyzed. The intrafraction uncertainty was defined as the dose volume histogram (DVH) difference between the two CBCT series. Thus we quantified the dosimetric uncertainty exclusively due to the intrafraction organ motion in relation to time. This value was obtained by calculating the difference between the volume receiving a fixed dose value in the CBCT pre‐ and in the CBCT post‐treatment (e.g., $V_{95\%}$(${\text{CBCT}}_{I}$) ‐ $V_{95\%}$(${\text{CBCT}}_{\text{II}}$). The analysis was carried out both in terms of absolute difference (i.e., module of the difference, without considering the direction) and in terms of difference with sign (positive and negative), in order to evaluate whether there is any systematic under‐ and overestimation.

In all cases, the organs were included into the CBCT axial field of view, except for six cases in which bladder was not entirely included.

For normally distributed data, the Pearson correlation coefficient and the Student's t‐test were used.

## III. RESULTS

The time of CBCTs varied between 4 and 16 min, with mean time being 7.3±0.7 min. When online co‐registration was done, the mean time was 8.6 min with a range of 6 to 16 min. Conversely, when off‐line co‐registration was done, the mean time was 5.8 min with a range of 4–10 minutes.

**Figure 1:**
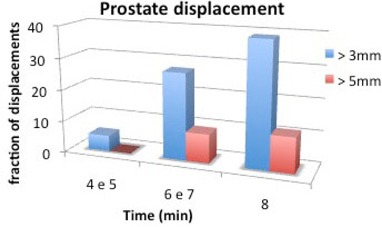
Percentage of patients with displacement greater than 3 and 5 mm as a function of time.

Table 1 reports the mean COM displacement for prostate, rectum and bladder. Poor correlation with elapsed time was found for prostate and rectum (r=0.67 and r=0.68). It was more significant for bladder (r=0.88, p=0.02 in the three cases). Considering the data stratified in four series according to the total treatment time, increasing average COM displacements and SDs were found, as shown in Table 1. In Fig. 1, the percentage of patients with displacement of > 3 mm and > 5 mm are reported as a function of increasing elapsed time. Contouring uncertainties by the double contouring in prostate, rectum and bladder were estimated to be 7%, 4% and 3%.

**Table 1:** Mean shift for rectum, bladder and prostate as a function of time.

		*Bladder*		*Rectum*		*Prostate*	
*Time (min)*	*Occurrence*	*Displ.(cm)*	*SD (cm)*	*Displ.(cm)*	*SD (cm)*	*Displ.(cm)*	*SD (cm)*
4–5	20	0.22	0.13	0.22	0.13	0.16	0.06
6–7	22	0.21	0.12	0.34	0.19	0.26	0.17
8	18	0.28	0.15	0.32	0.27	0.29	0.16
9–16	20	0.4	0.31	0.3	0.36	0.26	0.21

**Figure 2:**
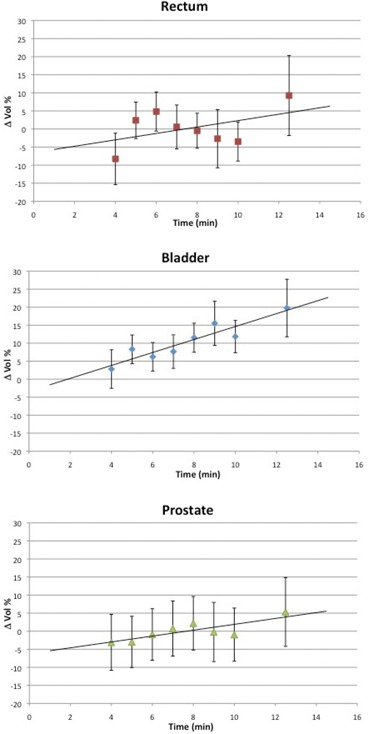
Mean volume variation for rectum, bladder and prostate as a function of time. The negative volume variation observed for the prostate is largely due to contouring uncertainties.

Concerning volume changes, an average increase of 10.6%±7.5% was found for the bladder, while this was of 3.2%±10.8% for rectum and −0.8%±6.6% for prostate. Figure 2 reports the volume differences related to time. A significant correlation was found for bladder and prostate: r=0.93 and 0.82, respectively. Conversely for rectum, a poor correlation was calculated: r=0.46. For these data, *p* was <0.3, 0.001 and 0.003, respectively. As expected, there are no significant variations in prostate volume pre‐ and post‐therapy; on the other hand, the volume variation of the bladder is to be ascribed to the filling of this organ with urine.

**Figure 3:**
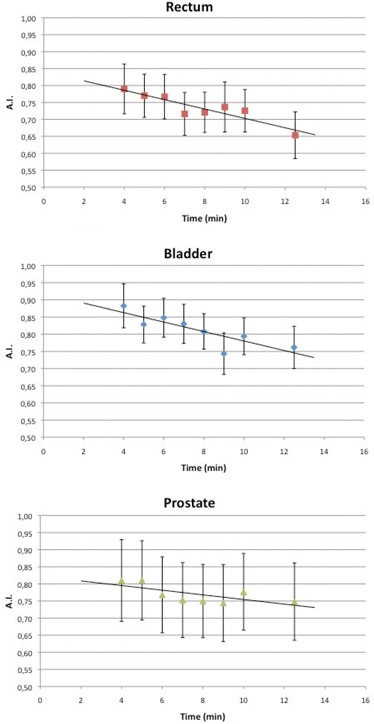
AI for rectum, bladder and prostate as a function of time.

Figure 3 reports the Agreement Index as a function of time. The coefficients were negative, indicating a decreasing overlap of the structures pre‐ and post‐treatment with increasing time: r=−0.89 for bladder, r=−0.95 for rectum and r=−0.84 for prostate. For all these data, p‐values were <0.01. This trend indicates an important dependence of the Agreement Index to therapy time and, therefore, this can be used to quantify volume variations and anatomic structures displacements during the course of treatment session.

**Figure 4:**
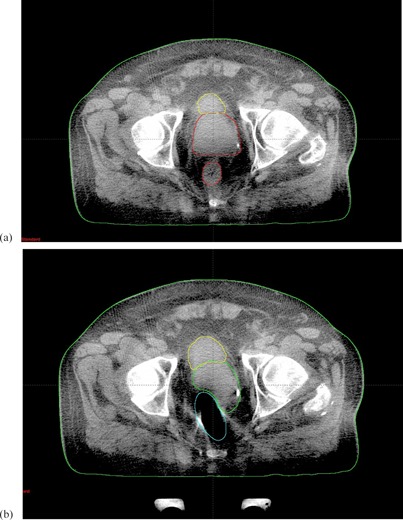
${\text{CBCT}}_{I}$ (a) and ${\text{CBCT}}_{\text{II}}$ (b) for a case of complete dislocation. The interval time was 6 min.

Three CBCT couples (i.e., 4%) were discarded from the analysis due to extraordinary air passage in rectum, two from patient 2 and one from patient 5. As an example, Fig. 4 shows that a severe rectum expansion was induced by a gas bubble transit during the treatment, inducing prostate dislocation. Image quality related data, or fast and transient phenomena as in this example, were excluded from the study, although the latter deserves further investigation. For these events, the statistics were too poor to perform a quantitative analysis and obtain any outcomes for this kind of event.

**Figure 5:**
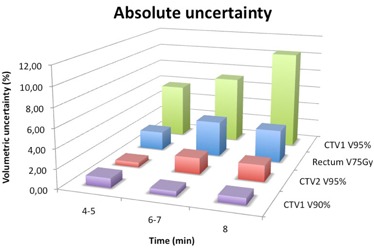
Absolute uncertainty for $V_{90\%}$ and $V_{95\%}$ of CTV1, $V_{95\%}$ of CTV2, and $V_{75\text{Gy}}$ of rectum.

Concerning the dose analysis, increasing uncertainties were found for high doses as shown in Fig. 5. We observed significant differences in dose between the first and the second CBCT and these uncertainties increase with treatment time. The dose differences were observed at the $V_{90\%}$ and $V_{95\%}$ points for CTV1, $V_{95\%}$ for CTV2 and the $V_{75\text{Gy}}$ point was investigated for rectum. Of particular interest is $V_{95\%}$ for CTV1 where dose uncertainty increases with time reaching 10% for an 8‐minute treatment. This means that even though patients can be repositioned before treatment, dose distribution is modified due to organ motion with variations whose extent increases with treatment time. This result is probably due to the fact that the treatment comprehends an integrated boost so that there is a steep dose gradient in correspondence of the seminal vesicles, where the most important displacements are observed. This hypothesis is confirmed by the very low uncertainties observed for $V_{90\%}$ with values that never exceed 1%.

**Figure 6:**
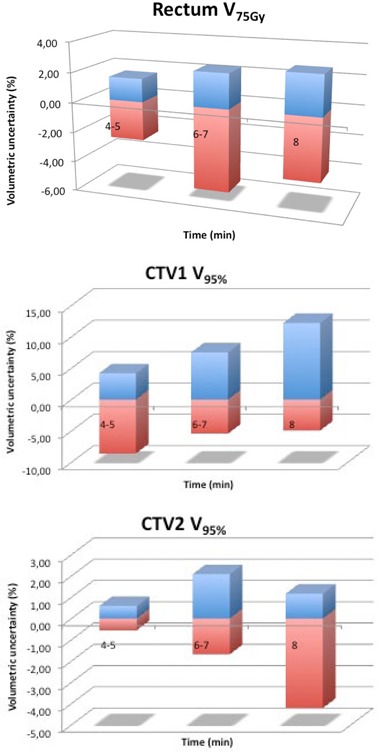
Relative uncertainty of rectum $V_{75\text{Gy}}$ (a), and for $V_{95\%}$ of CTV1 (b) and CTV2 (c). The positive values represent the mean overdosage and the negative values, the mean underdosage.

In Fig. 6, the uncertainties were separated in over‐ (positive values) and underdosages (negative values) for the same points of interested considered in the previous analysis. This distinction was made in order to observe whether the uncertainty observed in Fig. 5 had an over‐ or underdosage effect; actually, a time‐increasing underdosage for the seminal vesicles was observed.

## IV. DISCUSSION

Pelvic organ motion during treatment has been debated for a long time. The advent of highly conformant techniques (3D‐CRT and IMRT) on the one hand have improved the efficiency of radiotherapy treatments but, on the other hand, have made it much more important to quantify organ motion and evaluate its impact on treatment. Many authors^(^ ^13^ ^–^ ^15^ ^)^ have assessed noteworthy changes in volume and position of organs in this region, as confirmed by investigations based on CT studies. However, the need to move the patient from the linac room and the repositioning of the patient on the CT scanner bed bring about anatomic changes that potentially cause a bias in the quantitative aspect of the measured alterations. In this context, the recent introduction of CBCT has allowed for the acquisition of images without moving the patient from the treatment position and, therefore, without compromising the image quality.

In our study, the same radiation oncologist performed the contours on the simulation CT and on each CBCT in order to exclude interoperator uncertainty. Authors estimated intraoperator uncertainty using CT images to be 5%^(^ ^16^ ^)^ and 6.2%^(^ ^17^ ^)^ for CTV2. In our study, an uncertainty of 7% for CTV2 definition was estimated on CBCT. This error is slightly higher than what is reported in literature but is in any case comparable to those measured on CT images.

Nakagawa et al.^(^ ^3^ ^)^ compared the position of the prostate on pretreatment CBCT to the prostate position during the treatment over a period of six days for one patient, finding that the mean and standard deviation of displacements at the time of pretreatment versus the treatment position was consistent with zero. However, data from only one case were analyzed and thus no statistical conclusion can be drawn from a case report.

Studies using IMRT with fixed gantry beams^(^ ^4^ ^)^ found that in 41%–45% of cases the displacement was greater than 3 mm, while in 15%–19% of cases the displacement was found to be > 5 mm. In these studies, the average treatment time was around 15–20 minutes. In our study we observed a lower percentage of patients with a prostate shift of > 3 mm and of > 5 mm, as only 24% and 5% of fractions, respectively, were affected. Our data are obtained without real‐time tracking and this may slightly affect the results due to the possible underestimation of the displacements. However, the reliability of using the post‐treatment CBCT for the evaluation of the maximum displacement is assessed by Adamson in Adamson et al.^(^ ^4^ ^)^

Polat et al.^(^ ^18^ ^)^ analyzed intrafractional changes of prostate position during IMRT with a static field by CBCT pre‐ and post‐treatment. They did not find treatment time to be correlated with larger uncertainties in prostate dislocation. Conversely, our data showed a good correlation between time and AI. A reason for this discrepancy could be the time interval between the two CBCTs. In the Polat series, the time from the beginning of the first CBCT and the end of the post‐treatment CBCT was around 16 min. In our series, the treatment time was shorter: about 60 s of beam‐on to deliver the prescribed fraction of 2 Gy plus the imaging time, resulting in a mean time between pre‐ and post‐CBCT of ~7 min (i.e., less than 50% of the Polat series). To confirm our findings, displacements of 2–3 mm for prostate in AP direction were estimated by using internal fiducials for 2–7 min.^(^ ^19^ ^,^ ^20^ ^)^ These findings are lower with respect to the 6 mm evidenced by Polat. Furthermore, Langen et al.^(^ ^21^ ^)^ found that the percentage of single treatments with prostate displacement of > 3 mm to increase with time by using electromagnetic markers implanted in the prostate. Similar results were obtained in our series as shown in Fig. 1. Finally, Ghilezan et al.^(^ ^22^ ^)^ during a one‐hour MRI session acquired images of the prostate observing displacements for 11 points of interest. The calculation of the displacement probability showed an increase of this probability with time for all the points observed and the higher the displacement magnitude, the steeper was the increase. These results underline the importance of accounting treatment time in prostate radiotherapy.

The maximum volume variation observed in our series, considering all 160 CBCTs, were 56% to 110% for bladder and rectum, respectively. The observed increase in bladder filling was not correlated to the prostate position. These data are also confirmed in literature.^(^ ^18^ ^)^ As for the rectum, a volume increase of more than 50% was observed in three cases; this can lead to an unexpected target translocation, as shown in Fig. 4 and can compromise the daily session distribution. In a standard treatment of 35–40 fractions this can be neglected, but in the case of hypofractionation, this could lead to important uncertainties.

The following step was followed to evaluate how the intrafraction variability influences the dose distribution uncertainties between the pre‐treatment and post‐treatment CBCT. This dosimetric analysis regarding intrafraction uncertainty is the original contribution of this work, especially due to the fact that with VMAT we can investigate very short treatments and observe the time dependence of this uncertainty up to 3–4 minute treatments. For this purpose, the RapidArc plans optimized on the simulation CT were recalculated on the ${\text{CBCT}}_{I}$ and ${\text{CBCT}}_{\text{II}}$ scans. Yang et al.^(^ ^12^ ^)^ reported an accuracy lower than 2% by using the CBCT to evaluate dose distribution in prostate region, thus acceptable for our purpose. We analyzed the DVH of CTV1, CTV2 and rectum. For the bladder, the DVH curve was slightly better for superior times (data not shown), but this is due to its increasing volume, as the DVH is defined relative to the total volume of the organ that, in the case of bladder, increases constantly. Moreover, we focused our study on the lower limit of the two CTVs (i.e., $V_{95\%}$) to make sure the target has been fully covered during the daily treatment, and also on the high dose to rectum ($V_{75\text{Gy}}$) in order to estimate acute toxicity (bleeding). The steep dose gradients that IMRT techniques can achieve lead to non‐negligible dosimetric fallouts. These characteristics justify the great dosimetric differences observed between CTV1 uncertainties for $V_{90\%}$ and $V_{95\%}$. This fact stresses the importance of accounting for intrafraction variability, whereby time is an important parameter. Varadhan et al.^(^ ^13^ ^)^ found the maximum rectum variation that received the $V_{75\text{Gy}}$ to be 12%, but did not give any data regarding time dependence. In our study, we found a great dependence on time for both rectum and target.

All these results emphasize the need for limiting the field on time as much as possible in the pelvic area. In this context, RapidArc has shown to be feasible both in term of field on‐time and dose distribution, with high doses to the target volume and sparing of adjacent normal tissues.

Furthermore, these data revealed that the DVH constraints commonly accepted and used in RT practice could be influenced by intrafraction variability and, therefore, this should be included into DVH constraints definition.

## V. CONCLUSIONS

We quantified the intrafraction internal motion in pelvic region using pre‐ and post‐treatment CBCT. Using RapidArc, we investigated very short time intervals of up to 4 minutes (around one‐quarter of typical IMRT with fixed entrance time). We observed the percentage of fractions over 3 to 5 mm less than those reported for techniques with higher processing times. The discovery that time is strongly dependent on AI and on volume receiving $V_{75\text{Gy}}$ in the rectum and $V_{95\%}$ for CTV1 and CTV2, highlights the need to use RT techniques with a short delivery time.

## ACKNOWLEDGMENTS

The authors would like to thank Mr Antonio Modugno and all the radiographers and staff of the Radiotherapy Department, IRCCS Istituto Clinico Humanitas in Milan, for the support given in this study.
